# Supplementary material for: Risk-taking behaviors in adolescent men who have sex with men (MSM): An association between homophobic victimization and alcohol consumption
Source: PLoS One. 2021 Dec 2;16(12):e0260083. doi: 10.1371/journal.pone.0260083 (PMC8638971; doi:10.1371/journal.pone.0260083)
Supplement: S1 Table — (DOCX) [file pone.0260083.s001.docx]

**S1 Table. Percent change in odds ratio of multivariable models to assess change in the association between homophobic victimization and underage drinking among adolescent MSM.**

| **Forms of homophobic victimization** | **Odds Ratio***  **(Underage drinking)** | **Percent change** |
| --- | --- | --- |
| Exposure to at least one form of homophobic victimization | 2.02 | **0** |
| Verbally insulted (yelled at, criticized) | 1.99 | -1.5 |
| Someone threatened to out you | 1.28 | -36.6 |
| Threatened with physical violence | 1.59 | -21.3 |
| Been punched, kicked, or beaten | 1.41 | -30.2 |
| Attacked sexually | 1.65 | -18.3 |
| Your property was damaged | 1.63 | -19.3 |
| Been spat upon | 1.75 | -13.4 |
| Threatened with a knife, gun, or another weapon | 1.60 | -20.8 |

*Multivariable models adjusted for age, sexual orientation, mother’s education, father’s education, health literacy, race, and ethnicity
