# Supplementary material for: Risk-taking behaviors in adolescent men who have sex with men (MSM): An association between homophobic victimization and alcohol consumption
Source: PLoS One. 2021 Dec 2;16(12):e0260083. doi: 10.1371/journal.pone.0260083 (PMC8638971; doi:10.1371/journal.pone.0260083)
Supplement: S2 Table — (DOCX) [file pone.0260083.s002.docx]

**S2 Table. Percent change in odds ratio of multivariable models to assess change in the association between homophobic victimization and riding with an intoxicated driver or driving while under the influence among adolescent MSM.**

| **Forms of homophobic victimization** | **Odds Ratio***  **(Riding/ Driving while under the influence)** | **Percent change** |
| --- | --- | --- |
| Exposure to at least one form of homophobic victimization | 2.25 | 0 |
| Verbally insulted (yelled at, criticized) | 2.08 | -7.6 |
| Someone threatened to out you | 2.05 | -8.9 |
| Threatened with physical violence | 1.22 | -45.8 |
| Had an object thrown at you | 1.35 | -40.0 |
| Been punched, kicked, or beaten | 1.60 | -28.9 |
| Someone chased or followed you | 1.59 | -29.3 |
| Attacked sexually | 1.83 | -18.7 |
| Your property was damaged | 1.66 | -26.2 |
| Been spat upon | 1.75 | -22.2 |

*Multivariable models adjusted for age, sexual orientation, mother’s education, father’s education, health literacy, race, and ethnicity
