# Supplementary material for: Risk-taking behaviors in adolescent men who have sex with men (MSM): An association between homophobic victimization and alcohol consumption
Source: PLoS One. 2021 Dec 2;16(12):e0260083. doi: 10.1371/journal.pone.0260083 (PMC8638971; doi:10.1371/journal.pone.0260083)
Supplement: Attachment — Submitted filename: Review for Risk taking YMSM Victimization and Alcohol.pdf [file pone.0260083.s004.pdf]

The purpose of this study was to examine if there was an association between homophobic victimization and two alcohol related outcomes – alcohol consumption and riding with an intoxicated driver/riding in a car while under the influence of alcohol or drugs. The article is methodologically sound and has the potential to contribute a more nuanced understanding of how different forms of homophobic victimization contribute to substance use related outcomes for young men who have sex with men (YMSM). As the authors point out, there have been numerous studies that find this association--the novel contributions of this study are (1) looking at different aspects of homophobic victimization, (2) focusing on YMSM, and (3) looking at a less studied outcome (riding with an intoxicated driver or driving while intoxicated). My main, critique of the study is that these novel contributions are not highly emphasized, and the front end of the paper is highly general rather than specific to the current study, which is critical given numerous other studies with similar findings.

Abstract

Given the cross-sectional nature of the data, the authors should rephrase this sentence, "Adolescent men who experience homophobic victimization are at increased risk of alcohol consumption and risky-alcohol related behaviors." so that it sounds less directional.

Introduction

On page 5, the authors conclude by saying: "However, results have been inconsistent, including a null finding [24]. More research is needed to better understand risk factors contributing to underage drinking among SGM youth." Its unclear which results have been inconsistent – either that homophobic victimization is linked to alcohol related behaviors, or that school-based victimization (not clear if the school-based victimization was LGB specific or not) being the strongest predictor is inconsistent—either way, one null finding does not necessarily make the findings inconsistent given how much research has been done on both topics. The authors should consider rephrasing this sentence to provide a stronger conclusion regarding previous research, perhaps linking it specifically why more research is needed for homophobic victimization as a risk factor for underage drinking.

In the last paragraph before the methods the authors clearly outline the gaps of previous research, but do not provide much specific justification for *why* these particular gaps matter and are too general.

For the first gap, I do not buy that there is sparse research on homophobic victimization outside of school contexts—the Katz-Wise study that the authors cite, addresses several types of non-school-based victimization for LGB people in research studies conducted up to 2012 (which was 9 years ago), and school-based victimization was not the most frequent forms of victimization measured in those studies.

For the second gap—it is true that few studies compare several forms of homophobic victimization, but the authors should explain why comparing different types of homophobic victimization matters for understanding the effect of homophobic victimization on substance use.

For the third gap, there have been plenty of studies on risk factors within SGM groups, the statement is again very general, which make it boarder on being untrue—there are lots of studies on risk factors within SGM groups (MSM in particular), but there are fewer specific to homophobic victimization. The authors should spend more time addressing why this is important for the group they study, what will understanding this association among YMSM tell us that previous studies cannot or do not?

Lastly, one novel contribution of this study is looking at a less common substance use-related outcome-- riding with an intoxicated driver or driving while intoxicated—and the authors do not provide much rationale for examining this additional outcome.

Methods

The methods are solid. One question had that was unaddressed was how the authors dealt with missing data. There doesn't appear to be much missing on homophobic victimization, but there is some missing on some of the covariates would mean that the regressions would have different sample sizes.

I also noted here that there is a discrepancy with abstract where it says that 88% of youth reported at least one form of homophobic victimization, and in the text it is reported as 87% in text—"Most participants (87%) were exposed to at least one form of homophobic victimization in their lifetime"

Discussion

In the first full paragraph of the discussion, the authors mention several studies and prevalence rates of different types (homophobic?) victimization from other studies, but then conclude by saying, "Caution should be taken when comparing results across studies due to differences in the study populations and the measure of homophobic victimization." Why mention the prevalence rates then? What does this add to understanding the current study? This space could be better utilized discussing why these different associations inform our understanding risk factors for substance use-related behaviors.

For example, its perhaps less surprising that someone who was attacked sexually had greater odds of risky substance use behaviors because there is a research base supporting this association, but why having one's property damaged be related to substance use? Along the same lines, its notable that many of the odds ratios are pretty similar—actually, the largest association was for experiencing any victimization, which is not an uncommon way to use a multi-item measure homophobic victimization. So, do the different forms matter if they all have similar effects on the outcomes? Even if this is not discussed in this specific paragraph, this is not addressed at all throughout the discussion, yet it is one of the important and novel contributions of the study.
