# Supplementary material for: Risk-taking behaviors in adolescent men who have sex with men (MSM): An association between homophobic victimization and alcohol consumption
Source: PLoS One. 2021 Dec 2;16(12):e0260083. doi: 10.1371/journal.pone.0260083 (PMC8638971; doi:10.1371/journal.pone.0260083)
Supplement: Attachment — Submitted filename: Response to Reviewers.docx [file pone.0260083.s005.docx]

**Author’s response to reviewers' comments:**

**We thank the reviewers for their comments and suggestions on our manuscript, entitled “Risk-taking behaviors in adolescent men who have sex with men (MSM): An association between homophobic victimization and alcohol consumption.” We substantially revised our manuscript in response to these suggestions, and we feel that our revised manuscript is stronger because of these changes. Revisions are tracked in the revised manuscript. Detailed responses to reviewers are as follows (see bold font):**

**Reviewer #1**:

The data analysis is routine and includes univariate analyses with non parametric approaches such as Fisher’s exact test when appropriate. Logistic regression models and multivariable logistic regression were applied appropriately. The sample size appears reasonable. The conclusion appears to follow from the analysis in that adolescent men who experience homophobic victimization are at increased risk of alcohol consumption and risky-alcohol related behaviors.

1. The strengths and weaknesses of the manuscript are noted by the investigators. However, some concordance and sensitivity measures of these models should have been included in the analyses.

**Thank you for this suggestion. However, we believe that concordance measures are not appropriate to compare the models in our study because such measures are most often used to assess the discriminative ability of risk prediction models, whereas our analysis included causal models. Additionally, a sensitive analysis is used to compare models with varying modelling methods of analysis, different definitions of outcomes, or assess the effects of protocol deviations, missing data, or outliers. This was not the case in our study; all models used the same method of analysis and outcome definition but had different exposure variables (i.e., varies forms of victimization). Instead, to accurately compare these models to one another we compared the effect measures of each model with specific forms of victimization to the effect measure of the generalized victimization model (those experiencing any form of victimization). This analysis has been added as supplementary tables on page 25 of the manuscript.**

**Reviewer #2**:

1. The purpose of this study was to examine if there was an association between homophobic victimization and two alcohol related outcomes – alcohol consumption and riding with an intoxicated driver/riding in a car while under the influence of alcohol or drugs. The article is methodologically sound and has the potential to contribute a more nuanced understanding of how different forms of homophobic victimization contribute to substance use related outcomes for young men who have sex with men (YMSM). As the authors point out, there have been numerous studies that find this association--the novel contributions of this study are (1) looking at different aspects of homophobic victimization, (2) focusing on YMSM, and (3) looking at a less studied outcome (riding with an intoxicated driver or driving while intoxicated). My main, critique of the study is that these novel contributions are not highly emphasized, and the front end of the paper is highly general rather than specific to the current study, which is critical given numerous other studies with similar findings.

**Thank you for this comment. We have revised the language in third paragraph of the introduction to emphasize the novel contributions of this study to the current literature, as suggested.**

2. Abstract: Given the cross-sectional nature of the data, the authors should rephrase this sentence, “Adolescent men who experience homophobic victimization are at increased risk of alcohol consumption and risky-alcohol related behaviors.” so that it sounds less directional.

**This sentence has been revised to read: Increased risk of alcohol consumption and risky alcohol-related behaviors were found among adolescent men who experienced homophobic victimization.**

3. Introduction: On page 5, the authors conclude by saying: “However, results have been inconsistent, including a null finding [24]. More research is needed to better understand risk factors contributing to underage drinking among SGM youth.” Its unclear which results have been inconsistent – either that homophobic victimization is linked to alcohol related behaviors, or that school-based victimization (not clear if the school-based victimization was LGB specific or not) being the strongest predictor is inconsistent—either way, one null finding does not necessarily make the findings inconsistent given how much research has been done on both topics. The authors should consider rephrasing this sentence to provide a stronger conclusion regarding previous research, perhaps linking it specifically why more research is needed for homophobic victimization as a risk factor for underage drinking.

**Thank you for this suggestion. We have revised this sentence to read: Future research is needed to identify the specific forms of homophobic victimization experienced by SGM youth associated with increased risk of alcohol consumption for a comprehensive understanding of risk factors contributing to underage drinking in this population.**

4. Introduction: In the last paragraph before the methods the authors clearly outline the gaps of previous research, but do not provide much specific justification for why these particular gaps matter and are too general.

**Justification for studying specific homophobic victimization has been mentioned as: Such information is also essential to fully capture the homophobic victimization experiences of SGM youth and how these experiences contribute to the risk of underage drinking for the implementation of future interventions.**

**Justification for studying adolescent MSM only has been mentioned as: Research on underage alcohol consumption among adolescent MSM is needed to improve our understanding of alcohol-related disparities found within the SGM population.**

**Justification for study the association between homophobic victimization and driving while intoxicated or riding with an intoxicated driver has been added to the manuscript as: Previously, over 20% of SGM youth reported riding with an intoxicated driver and 8% drove while intoxicated [9]. Identifying homophobic victimization as a risk factor for alcohol-related behaviors may inform future studies and provide evidence that victimization can influence adverse health behaviors beyond substance abuse, and significantly impact on the health of adolescent MSM.**

5. Introduction: For the first gap, I do not buy that there is sparse research on homophobic victimization outside of school contexts—the Katz-Wise study that the authors cite, addresses several types of non-school-based victimization for LGB people in research studies conducted up to 2012 (which was 9 years ago), and school-based victimization was not the most frequent forms of victimization measured in those studies.

**The first gap mentioned has been removed from the introduction, as suggested.**

6. Introduction: For the second gap—it is true that few studies compare several forms of homophobic victimization, but the authors should explain why comparing different types of homophobic victimization matters for understanding the effect of homophobic victimization on substance use.

**As suggested, we added a sentence to emphasize the importance of studying several forms of homophobic victimization. This sentence reads: By identifying which form of homophobic victimization is associated with alcohol consumption, targeted interventions can be implemented to end specific victimization tactics aimed at SGM youth.**

7. Introduction: For the third gap, there have been plenty of studies on risk factors within SGM groups, the statement is again very general, which make it boarder on being untrue—there are lots of studies on risk factors within SGM groups (MSM in particular), but there are fewer specific to homophobic victimization. The authors should spend more time addressing why this is important for the group they study, what will understanding this association among YMSM tell us that previous studies cannot or do not?

**As suggested, we added a few sentences to address why it is important to study the association between homophobic victimization and alcohol consumption among YMSM. These sentences read:** **Second, little research has been conducted to understand victimization as a risk factor for alcohol use specifically for adolescent men who have sex with men (MSM) [3]. We contend that adolescent MSM may be at particularly high risk of underage drinking due to the severe forms of victimization more often reported by them compared to other SGM subgroups [18],[20], warranting concern and future research. Additionally, by focusing on adolescent MSM alone, researchers will be able to compare alcohol consumption among adolescent MSM who experienced homophobic victimization to adolescent MSM who did not, whereas previous comparisons have been made to heterosexual youth [2], [6], [8].**

8. Introduction: Lastly, one novel contribution of this study is looking at a less common substance use-related outcome-- riding with an intoxicated driver or driving while intoxicated—and the authors do not provide much rationale for examining this additional outcome.

**We have revised the language in the introduction to provide more rationale for why we examined the outcome ‘riding with an intoxicated driver or driving while intoxicated’. We added the following sentences: Third, to our knowledge, no previous study has assessed the association between homophobic victimization and risky alcohol-related behaviors, specifically driving while intoxicated or riding with an intoxicated driver. Previously, over 20% of SGM youth reported riding with an intoxicated driver and 8% drove while intoxicated [9]. Identifying homophobic victimization as a risk factor for alcohol-related behaviors may inform future studies and provide evidence that victimization can influence adverse health behaviors beyond substance abuse, and significantly impact on the health of adolescent MSM.**

9. Methods: The methods are solid. One question had that was unaddressed was how the authors dealt with missing data. There doesn’t appear to be much missing on homophobic victimization, but there is some missing on some of the covariates would mean that the regressions would have different sample sizes.

**Thank you for pointing out this omission. We added the sentence “Participants with missing data on covariates were dropped from the multivariable regression models” to clarify how missing data was handled in the regression models on page 9, line 199-200.**

10. Methods: I also noted here that there is a discrepancy with abstract where it says that 88% of youth reported at least one form of homophobic victimization, and in the text it is reported as 87% in text—"Most participants (87%) were exposed to at least one form of homophobic victimization in their lifetime”

**This sentence has been corrected in the abstract and now reads:** **Most participants (87%) reported at least one form of homophobic victimization in their** **lifetime, with verbal insults being the most frequently reported (82%). This reflects the data presented in text and in Table 2.**

11. Discussion: In the first full paragraph of the discussion, the authors mention several studies and prevalence rates of different types (homophobic?) victimization from other studies, but then conclude by saying, “Caution should be taken when comparing results across studies due to differences in the study populations and the measure of homophobic victimization.” Why mention the prevalence rates then? What does this add to understanding the current study? This space could be better utilized discussing why these different associations inform our understanding risk factors for substance use-related behaviors. For example, its perhaps less surprising that someone who was attacked sexually had greater odds of risky substance use behaviors because there is a research base supporting this association, but why having one’s property damaged be related to substance use? Along the same lines, its notable that many of the odds ratios are pretty similar—actually, the largest association was for experiencing any victimization, which is not an uncommon way to use a multi-item measure homophobic victimization. So, do the different forms matter if they all have similar effects on the outcomes? Even if this is not discussed in this specific paragraph, this is not addressed at all throughout the discussion, yet it is one of the important and novel contributions of the study.

**Thank you for this comment. The first full paragraph on prevalence rates has been removed from the discussion, as suggested. A discussion on the different associations and how they inform our understanding of risk factors for substance use-related behaviors has been added on page 20, line 319‒331. We also addressed the finding that the different forms of victimization have similar effect on the outcomes in order to increase clarity of its significance.**
