# Supplementary material for: Risk-taking behaviors in adolescent men who have sex with men (MSM): An association between homophobic victimization and alcohol consumption
Source: PLoS One. 2021 Dec 2;16(12):e0260083. doi: 10.1371/journal.pone.0260083 (PMC8638971; doi:10.1371/journal.pone.0260083)
Supplement: Attachment — Submitted filename: Response to Reviewers #2.docx [file pone.0260083.s006.docx]

**Editor and Reviewer comments:**

**We thank the reviewers for their comments and suggestions on our manuscript, entitled “Risk-taking behaviors in adolescent men who have sex with men (MSM): An association between homophobic victimization and alcohol consumption.” We substantially revised our manuscript in response to these suggestions, and we feel that our revised manuscript is stronger because of these changes. Revisions are tracked in the revised manuscript. Detailed responses to reviewers are as follows (see bold text below):**

**Review Comments to the Author**

Please use the space provided to explain your answers to the questions above. You may also include additional comments for the author, including concerns about dual publication, research ethics, or publication ethics. (Please upload your review as an attachment if it exceeds 20,000 characters)

Reviewer #1: (No Response)

Reviewer #2: The authors have generally responded well to my comments. I have a few remaining suggestions listed below.

**Reviewer #2 Comments:**

1. I recommend that the authors update their literature review and focus it only on research specific to MSM or SGM men. Much of the research on SGM populations related to alcohol use and substance use is discussed in terms of gender difference among SGM populations, so general findings about SGM don’t necessarily apply to this study focused on MSM. I have more specific suggestions below, but the discussion should also be focused more around MSM than SGM populations more generally.

**Thank you for this thoughtful comment. While we agree with the reviewer that much of the research on SGM populations related to alcohol use and substance use is discussed in terms of gender difference among SGM populations, our literature review was limited by the current lack of research on alcohol use among adolescent MSM. Because this study focused on underage drinking among adolescent MSM, we believe that a comparison to older MSM (i.e., 21 years or older) may not highlight the risk of this behavior. Instead, we aimed to compare our results to other youth who may be at risk of underage drinking (i.e., SGM youth).**

2. I noted some typographical errors in the literature review, so the authors should carefully edit the manuscript.

**We thoroughly reviewed the full manuscript for typographical errors. The appropriate edits have been made to the manuscript.**

3. The authors have a claim that school-based homophobic victimization is one of the strongest predictors of alcohol use on page 5 line 95 and 96. However, neither of the references cited makes this claim and I am unsure that that there has been a study testing this claim. I recommend the authors remove the statement or update to say that school-based victimization has been found to be strongly related to substance use.

**As suggested by the reviewer, we revised the sentence to read “In fact, some studies have found school-based victimization to be strongly related to substance use among SGM youth [3], [15].”**

4. The discussion still has track changes in it.

**We apologize for this oversight. For this submission, we submitted two versions of the manuscript: 1. Manuscript with tracked changes and 2. Manuscript with no tracked changes (clean version).**

5. In the discussion, the authors state: “We found that half of the adolescent MSM study participants reported alcohol consumption, which is noteworthy since they are all under the US legal drinking age of 21. However, this estimate was lower than that previously reported by a nationwide study, where 75% of SGM high school students drank alcohol [9]. This difference may be explained by our younger sample population, which included both middle school and high school students.” This statement should be removed—the comparison is MSM to SGM which while there is overlap are not the same populations, so there are numerous reasons that the prevalence rates could differ beyond those the author’s mention, namely that SGM women tend to have more elevated rates of alcohol use than SGM men.

**Thank you for your suggestion, this statement has been removed.**

6. In the discussion, the authors state: “We found that among adolescent MSM underage drinking was associated with various forms of homophobic victimizations, such as verbal insults or threats, physical or sexual assaults, and property damage. This finding aligned with results from existing studies focused on school-victimization and alcohol use among SGM youth.” It is unclear why they compare their results to studies on school victimization given that their study focuses on general victimization of MSM.

**This is a good point made by the reviewer. While we agree with the reviewer that our study focuses on general victimization among adolescent MSMS, the results of our study were compared to studies on school-based victimization because this is the data currently available. We revised this sentence to read, “We compared our results to studies focused on school-based victimization and alcohol use among SGM youth due to the lack of data surrounding homophobic victimization in MSM youth in other setting.”**
